# Developing Further Versatility in Benzoxazine Synthesis via Hydrolytic Ring-Opening

**DOI:** 10.3390/polym12030694

**Published:** 2020-03-20

**Authors:** Shaoying Cui, Carlos R. Arza, Pablo Froimowicz, Hatsuo Ishida

**Affiliations:** 1 State Key Laboratory of Polymer Materials Engineering, Polymer Research Institute of Sichuan University, Chengdu 610065, China; cuishaoying1993@gmail.com; 2 Department of Macromolecular Science and Engineering, Case Western Reserve University, Cleveland, OH 44106-7202, USA; caroarza@gmail.com (C.R.A.); pxf106@case.edu (P.F.); 3 Design and Chemistry of Macromolecules Group, Institute of Technology in Polymers and Nanotechnology (ITPN), UBA-CONICET, FADU, University of Buenos Aires, Intendente Güiraldes s/n, Pabellon III, subsuelo, Ciudad Universitaria (P.C. C1428EGA), Buenos Aires C1428EGA, Argentina

**Keywords:** 2-substituted 1,3-benzoxazine, ring opening, HCl hydrolysis

## Abstract

In this study, 2-(aminomethyl)phenol and its derivatives, the reactants for 2-substituted 1,3-benzoxazines, are synthesized by HCl hydrolysis from the typical benzoxazines. The phenol/aniline-based mono-oxazine benzoxazine, PH-a, and the bisphenol A/aniline-based bis-oxazine benzoxazine, BA-a, are used as examples to demonstrate the feasibility of this new approach. Their chemical structures are characterized by nuclear magnetic resonance (NMR), Fourier transform infrared (FTIR) and Raman spectroscopies, and are further verified by elementary analysis. Their thermal properties are studied by differential scanning calorimetry (DSC). These two 2-(aminomethyl) phenolic derivatives are reacted with paraformaldehyde to close the oxazine rings. A benzoxazine with a phenyl substituent at the 2-position of the oxazine ring is obtained from the 2-((phenylamino)methyl)phenol (*h*PH-a) and benzaldehyde. All these results highlight the success of the HCl hydrolysis and the formation of stable intermediates, namely 2-(aminomethyl) phenolic derivatives, from readily available benzoxazine monomers. This further demonstrates the feasibility of using these intermediates as reactants for a novel benzoxazine synthesis.

## 1. Introduction

As a new class of thermosets, polybenzoxazines have drawn great attention from academic as well as industrial communities for their outstanding characteristics compared with other thermosets, such as epoxies, phenolics, bismaleimides and even polyimides. Notable advantages include their remarkable molecular design flexibility [1,2,3,4,5,6,7], excellent thermal and mechanical properties [8], high char yield [9], excellent flame retardancy [10,11,12,13,14], autocatalysis for ring-opening polymerization [15], near-zero shrinkage upon polymerization [16,17], excellent electrical properties [18,19,20,21,22,23,24,25] and surface energy lower than polytetrafluoroethylene [26,27,28,29,30].

In the last few decades, a great variety of benzoxazine monomers have been synthesized from a versatile condensation route, based on the Mannich reaction, from the combination of a phenolic derivative, formaldehyde, and a primary amine. The benzoxazine monomers show the excellent characteristic of molecular design flexibility due to the simplicity and diversity of the substituents on the phenol and amine compounds, and thus a wide range of reactants with different functional groups, such as the alkyl, alkenyl, aryl, halogen, cyano, aldehyde, maleimide, nitro, furan and carboxylic groups, have been employed to obtain benzoxazines for their desired properties and potential applications [1,2,3,4,5,6,7,31]. 

1,3-benzoxazine with substitutions on the C2 of its oxazine rings selectively (2-substituted 1,3-benzoxazine) is different from the traditional benzoxazines, which gives researchers a novel way to explore benzoxazines with new structures, functional groups and properties as polymers for potential applications. However, only a few papers reported 2-substituted 1,3-benzoxazine for other potential applications rather than thermoset materials. Two methods for preparing 2-substituted 1,3-benozxazine are reported. The rhodium-catalyzed reaction of 2-allyoxybenzylamines in the presence of H_2_/CO is utilized in the first method to obtain 2-substituted 1,3-benzoxazines [32,33], however, the reducing atmosphere is highly explosive, which is very dangerous in the laboratory, and the variety of 2-allyoxybenzylamine and its derivatives is limited. In recent years, the other more widely used procedure is to adopt salicylaldehyde and aniline to synthesize an intermediate Schiff base, followed by a reduction in sodium borohydride (NaBH_4_) and a ring-closure reaction with aldehyde, to obtain the 2-substituted 1,3-benzoxazines. A series of 2-substituted 1,3-benzoxazines by this method was reported by Tang et al. to evaluate their fungicidal activities in their monomer forms [34,35,36]. Ishida’s group studied the effect of oxazine ring substitution on the polymerization behavior, and on their properties in their polymer forms for the first time [37]. However, this synthesis method is redundant and time-consuming, and the number of available raw materials, which must possess an aldehyde group at the ortho-position with respect to the phenolic hydroxyl group, is highly limited. In addition, the cost of these compounds, especially the toxic NaBH_4_, is relatively high. All these reasons make this synthesis method unattractive, particularly for scale-up purposes. Therefore, a method that possesses simplification and versatility, and which is safer for the environment than this three-step synthesis, is desired. The proposed new method is attractive as it eliminates the use of NaBH_4_ and, even more importantly, it adds significant versatility to the design of new benzoxazine resins.

By analyzing the process, it is noticed that 2-(aminomethyl)phenol and its derivatives are the most important intermediates needed to obtain 2-substituted 1,3-benzoxazines. The formation of such a compound was reported in the literature, though no detailed analysis of the compound was given [38,39]. Thus, it is essential to simplify the steps taken to prepare this reduced intermediate. As discussed above, many 1,3-benzoxazines with different functional groups and properties have been successfully synthesized by the common Mannich condensation method in recent decades. Further, several benzoxazines have already been commercialized against great odds for the commercialization of newly developed polymers. In the last 40 years, only a few new polymers have been commercialized due, in part, to severe regulatory rules and stiff competition from the existing commercial polymers. If it is possible to utilize these commercially available benzoxazines as the raw materials needed to prepare the aforementioned reduced intermediate, not to mention those large numbers of laboratory-prepared benzoxazines, it is possible to open a path to further the vast number of 2-position substituted benzoxazines. These oxazine-ring-substituted benzoxazines have already been shown to polymerize, in addition to already exhibiting a great variety of common benzoxazines whose -CH_2_- portions of the oxazine ring are not substituted. Therefore, it is one of the goals of this current paper to design a methodology to develop an important intermediate using more well-known and environmentally benign conditions than the currently available methods so that the benzoxazine resins can be readily and safely synthesized.

In this paper, the oxazine rings of the typical phenol/aniline-based mono-oxazine benzoxazine (PH-a) and the bisphenol A/aniline-based bis-oxazine benzoxazine (BA-a) are opened by hydrolysis with HCl, and the structures of these two intermediates via a simple hydrolysis route are reported for the first time; however, a similar aminophenolic compound is synthesized using a multistep synthesis [40]. Nuclear magnetic resonance (^1^H NMR and ^13^C NMR) spectroscopy, Fourier transform infrared spectroscopy (FTIR), Raman spectroscopy and elementary analysis are used to characterize the structure of the intermediates, while differential scanning calorimetry (DSC) is employed to study their thermal properties. Then, these intermediates are used with formaldehyde to resynthesize PH-a and BA-a to confirm the feasibility of using these intermediates as independent compounds to synthesize benzoxazines. The ultimate goal of this study is to substitute formaldehyde with other forms of aldehydes, such as acetaldehyde, benzaldehyde and glutaraldehyde, to expand the synthesis of benzoxazines into further versatile compounds that allow for the tailoring of the polymers with desired properties, using the stable intermediates derived from readily available benzoxazine monomers.

## 2. Experimental

### 2.1. Materials

Phenol (≥99%), bisphenol A, magnesium sulfate anhydrous and sodium hydroxide were purchased from Sigma-Aldrich. Aniline (99%), toluene (99.9%), hydrochloric acid (37.5%), ammonium hydroxide (29% solution), hexane, ethyl acetate and acetone were purchased from Fisher Scientific. Dimethylsulfoxide-d was obtained from Cambridge Isotope Laboratories, Inc.

### 2.2. Monomer Synthesis

#### 2.2.1. Synthesis of 3-Phenyl-3,4-dihydro-2H-benzo[e] [1,3]-oxazine (abbreviated as PH-a)

Synthesis of PH-a was achieved by the reported method [41].

#### 2.2.2. Synthesis of 6,6’-(propane-2,2-diyl)bis(3-phenyl-3,4-dihydro-2H-benzo[e][1,3]oxazine) (abbreviated as BA-a)

Synthesis of BA-a was achieved by the reported method [42].

#### 2.2.3. Synthesis of 2-((phenylamino)methyl)phenol (abbreviated as hPH-a)

PH-a (500 mg) was dissolved in 15 mL of n-propanol, then hydrochloric acid (37.5% *w*/*w*, 7 mL) and distilled water (3.5 mL) were added to the solution. The mixture was magnetically stirred and heated under reflux for 2 h. After cooling to 0 °C, 29% ammonium hydroxide (15 mL) was added to the solution. The mixture was magnetically stirred for 1 h at room temperature. After extraction with ethyl acetate three times, the ethyl acetate solution was washed three times with water. The organic phase was dried in a vacuum oven. The resulting crude product was recrystallized three times from hexane/ethyl acetate (8/2) to obtain white crystals. Yield 92%. ^1^H NMR (600 MHz, DMSO-d_6_, ppm): δ 4.13 (d, 2H, Ar-CH_2_-N), 5.91 (t, 1H, -NH), 6.45-7.14 (9H, Ar-H), 9.44 (s, 1H, -OH). ^13^C-NMR (600 MHz, DMSO-d_6_, ppm): δ 41.7 (Ar-CH_2_-N), 112.6, 115.2, 116.0, 119.2, 126.2, 127.8, 128.6, 129.2, 149.2, 155.4. FTIR spectra (cm^−1^): 3261 (stretching vibration of-OH and -NH groups), 1597 (typical aromatic skeletal vibration), 1495 (stretching of disubstituted benzene ring), 796 (N-H out-of-plane bending vibration). 

#### 2.2.4. Synthesis of 4,4’-(propane-2,2-diyl)bis(2-((phenylamino)methyl)phenol) (abbreviated as hBA-a)

BA-a (500 mg) was dissolved in 20 mL of acetone, then hydrochloric acid (37.5% *w*/*w*, 10 mL) and distilled water (5 mL) were added to the solution. The mixture was magnetically stirred and heated under reflux for 2 h. After cooling to 0 °C, 29% ammonium hydroxide (20 mL) was added to the solution. The mixture was magnetically stirred for 1 h at room temperature. After extraction with ethyl acetate three times, the ethyl acetate solution was washed three times with water. The organic phase was dried in a vacuum oven. The dried viscous product was dissolved in ethyl acetate followed by filtration to obtain undissolved white powder. Then, the white powder was recrystallized from acetone three times to obtain white crystals. Yield 90.5%. ^1^H NMR (600 MHz, DMSO-d_6_, ppm): δ 1.37 (s, 6H, -CH_3_), 4.02 (d, 2H, Ar-CH_2_-N), 5.73 (t, 1H, -NH), 6.44-7.02 (9H, Ar-H), 9.22 (s, 1H, -OH). ^13^C-NMR (600 MHz, DMSO-d_6_, ppm): δ 31.3 (-CH_3_), 41.3 (Ar-C-CH_3_), 42.5 (Ar-CH_2_-N), 112.7, 114.7, 116.0, 124.9, 126.0, 127.3, 129.1, 141.2, 149.4, 153.1. FTIR spectra (cm^−1^): 3328 (stretching vibration of -NH groups), 3233 (stretching vibration of -OH groups), 1602 (typical aromatic skeletal vibration), 1497 (stretching vibration of trisubstituted benzene ring), 775 (N-H out-of-plane bending vibration).

#### 2.2.5. Resynthesis of PH-a and BA-a from hPH-a and hBA-a

Paraformaldehyde (45.2 mg) and *h*PH-a (300 mg) were added into a round-bottom flask to dissolve in toluene (5 mL). The mixture was stirred for 5 h under reflux. After the reaction, the solution was washed three times with 1 N sodium hydroxide aqueous solution and water until the pH reached 7. The organic phase was dried over magnesium sulfate and then further dried under a vacuum. The resulting product was recrystallized in hexane three times to afford PH-a crystals. Yield 93%. ^1^H NMR (600 MHz, CDCl3, ppm): δ 4.62 (s, 2H, Ar-CH_2_-N, 5.35 (s, 2H, O-CH_2_-N), 7.26-6.79 (9H, Ar-H). ^13^C-NMR (600 MHz, CDCl_3_, ppm): δ 50.4 (Ar-CH_2_-N), 79.5 (O-CH_2_-N), 116.9, 118.3, 120.8, 121.4, 126.7, 127.8, 129.3, 148.4, 154.4. FTIR spectra (cm^−1^): 1600 (typical aromatic skeletal vibration), 1491 (disubstituted benzene ring), 1222 (antisymmetric C-O-C stretching vibration), 934 (O-C_2_ vibration in the oxazine ring with a minor contribution from the phenolic ring), 753 (Ar-H out-of-plane vibration).

Paraformaldehyde (41.1 mg) and *h*BA-a (300 mg) was reacted in toluene (5 mL) by the same process to obtain crude BA-a, then the crude product was recrystallized three times by a toluene/acetone (3/1) mixed solvent to obtain pure BA-a crystals. Yield 86%. ^1^H NMR (600 MHz, CDCl_3_, ppm): δ 1.56 (s, 6H, -CH_3_), 4.57 (s, 4H, Ar-CH_2_-N), 5.32 (s, 4H, O-CH_2_-N), 7.26-6.68 (16H, Ar-H). ^13^C-NMR (600 MHz, CDCl_3_, ppm): δ 31.0 (-CH_3_), 41.8 (Ar-C-CH_3_), 50.1 (Ar-CH_2_-N), 79.1 (O-CH_2_-N), 116.4, 118.0, 120.1, 121.2, 124.7, 126.4, 129.2, 143.2, 148.5, 152.2. FTIR spectra (cm^−1^): 1600 (typical aromatic skeletal vibration), 1495 (trisubstituted benzene ring), 1231 (antisymmetric C-O-C stretching vibration), 950 (O-C_2_ vibration in the oxazine ring with a minor contribution from the phenolic ring), 761 (Ar-H out-of-plane vibration).

#### 2.2.6. Synthesis of 2,3-diphenyl-3,4-dihydro-2H-benzo[e][1,3]oxazine ((abbreviated as PH-a-[2]ba)

Benzaldehyde (159.8 mg) and *h*PH-a (300 mg) were stirred in toluene (5 mL) under reflux for 6 h in a flask. After the reaction, the solution was washed three times with 1 N sodium hydroxide aqueous solution and water until the pH reached 7. Then, the solution was dried over magnesium sulfate and stayed under a vacuum to evaporate the solvent. The crude product was recrystallized by hexane to afford colorless crystals. Yield: 82%. ^1^H NMR (600 MHz, CDCl_3_, ppm): 4.31(s, 2H, Ar-CH_2_-N), 6.63 (s, 1H, O-CHAr-N), 6.80-7.51 (m, 14H, Ar-H).

### 2.3. Characterization

The ^1^H and ^13^C nuclear magnetic resonance (NMR) spectra were acquired on a Varian Oxford AS600 at a proton frequency of 600 MHz and at a carbon frequency of 150.864 MHz. The average number of transients for the ^1^H and ^13^C NMRs was 16 and 256, respectively. A contact time of 10s was used for the quantitative analysis of the ^1^H NMR spectra. A Fourier transform infrared spectroscopic analysis (FTIR) was conducted on an MB 3000 series spectrophotometer (ABB Inc., Saint Laurent, QC Canada). The samples were ground with KBr powder and then compressed into tablets. The spectra were co-added by 32 scans at a resolution of 4 cm^−1^. The Raman spectra of the intermediate crystals were obtained at room temperature by a micro-Raman spectrophotometer (Horiba Jobin Yvon LabRam HR800 spectrometer) equipped with a charge-coupled detector and two grating systems (600 and 1800 lines/mm). A HeNe laser (=632.8 nm) with an optical power of 17 mW was focused on the intermediate single crystals using an Olympus microscope into a spot size of 1 m in diameter. Raman shifts were calibrated with a silicon wafer using the 520 cm^−1^ Raman line. An elemental analysis for the C, N, and H was performed at Galbraith Laboratories and was compared with the theoretically calculated value of the 100% pure compound. The samples were dried at 60 °C under a vacuum for 24 h to avoid moisture contributing to the error in the hydrogen concentration before the analysis was performed under an oxygen atmosphere. The TA Instruments DSC model 2920 was used with a heating rate of 10 °C/min and a nitrogen flow rate of 60 mL/min for the differential scanning calorimetric (DSC) study. 

## 3. Results and Discussion

**Scheme 1:**
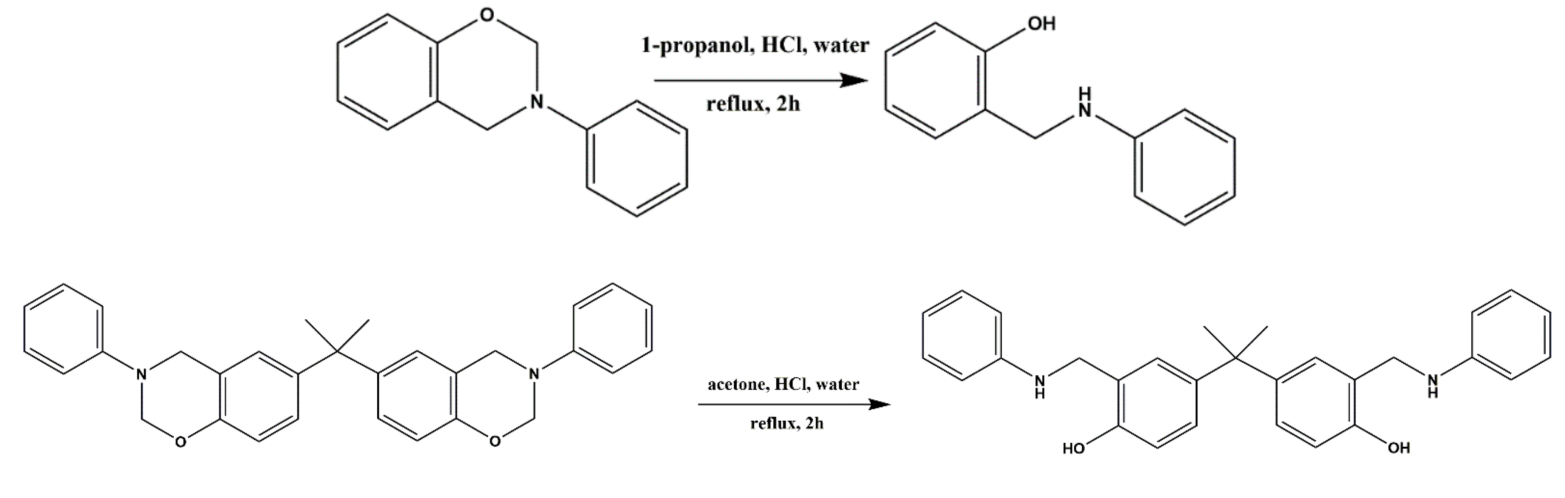
Synthetic scheme of *h*PH-a and *h*BA-a based on PH-a and BA-a.

Synthesis of these two 2-(aminomethyl) phenolic derivatives from PH-a and BA-a are shown in Scheme 1.

**Figure 1:**
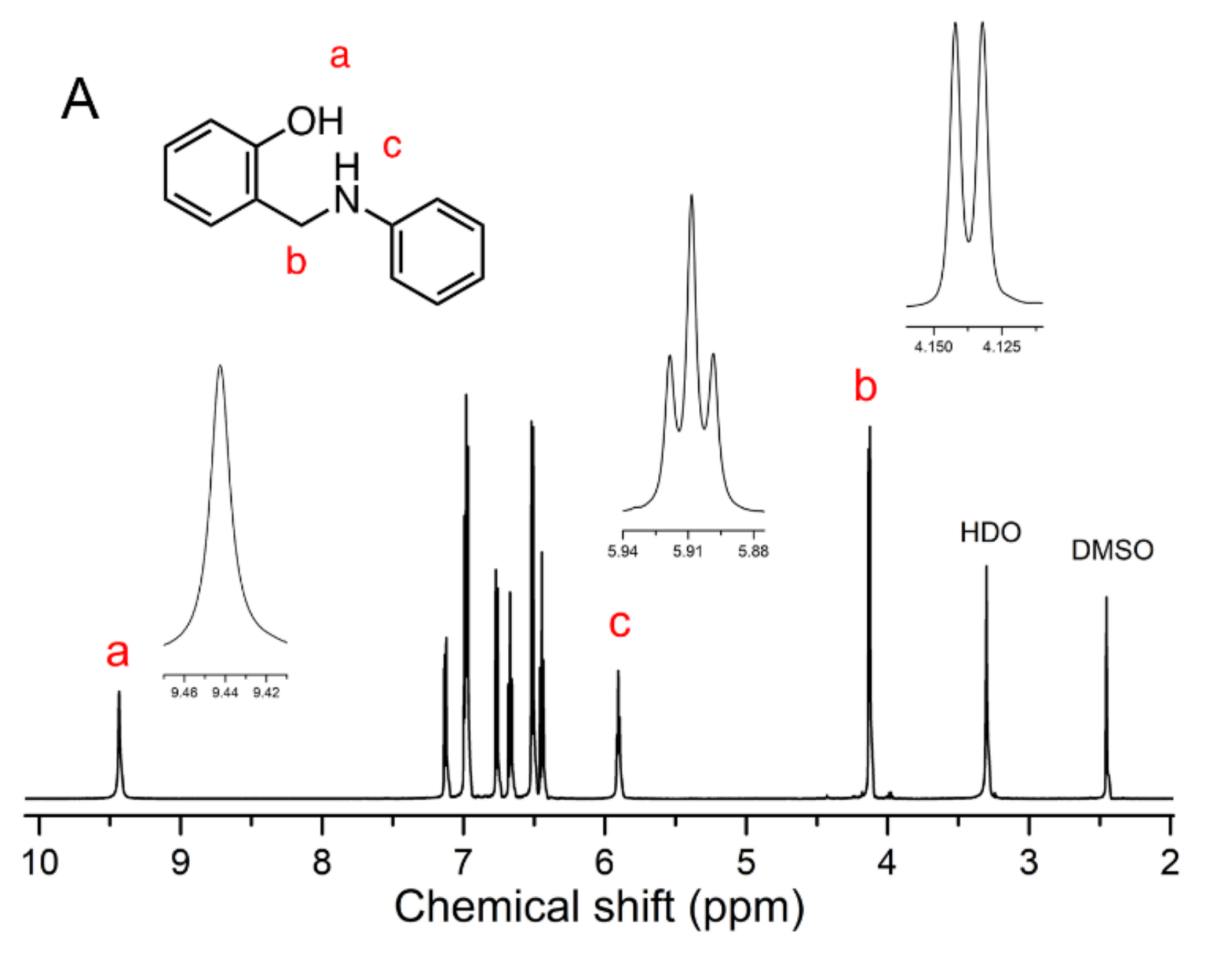

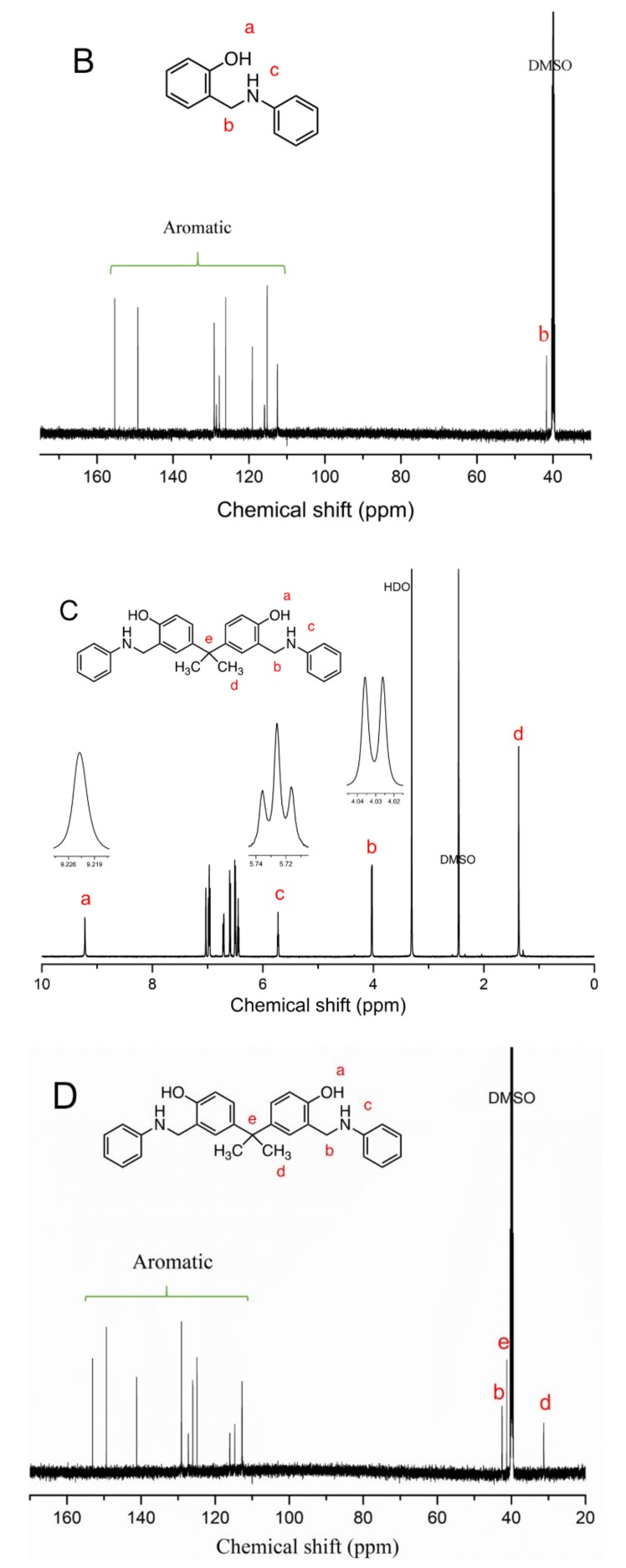
^1^H and ^13^C nuclear magnetic resonance (NMR) spectra of *h*PH-a (**A**,**B**) and *h*BA-a (**C**,**D**) in DMSO-d_6._

The chemical structures of the successfully hydrolyzed PH-a, *h*PH-a, and the hydrolyzed BA-a, *h*BA-a, are analyzed by the ^1^H and ^13^C NMRs, as shown in Figure 1. The opening of the oxazine ring at O-CH_2_-N gives rise to the -OH and -NH- groups, whose protons are observed at 9.47 ppm (a in Figure 1A) and 5.94 ppm (c in Figure 1A) as singlet and triplet resonances for *h*PH-a, respectively. Compared with PH-a, the Ar-CH_2_-N resonance in the open Mannich group of *h*PH-a shifts from 4.62 ppm to a lower value of 4.12 ppm (b in Figure 1A) because of the increased density of electron clouds in the changed chemical environment, and this peak splits into doublet resonances. As expected, the integrated intensities of the 9.47 and 5.94 ppm resonances are half of the 4.12 ppm resonance, indicating the presence of the single protons of -OH and -NH, respectively. Additionally, the ^13^C NMR spectrum shows one characteristic resonance assigned to the Ar-CH_2_-N carbon at 41.7 ppm (b in Figure 1B), which is also lower than that of PH-a (50.4 ppm). Meanwhile, the resonance at 79.5 ppm which is assigned to the O-CH_2_-N carbon of the oxazine ring disappears, confirming the successful opening of the oxazine ring. 

In the ^1^H NMR spectra of *h*BA-a, the same peak pattern of the -OH and -NH resonances as in *h*PH-a appear at 9.22 and 5.73 ppm (a, c in Figure 1C), respectively, while the Ar-CH_2_-N resonance decreases to 4.03 ppm (b in Figure 1C). In the ^13^C NMR, it is easy to recognize the resonance of Ar-CH_2_-N, >C< and -CH_3_ at 42.5, 41.3 and 31.3 ppm (b, c and d in Figure 1D), respectively. The resonances of O-CH_2_-N were not observed in both the ^1^H and ^13^C NMR spectra, reflecting the successful opening of the oxazine ring of BA-a.

**Figure 2:**
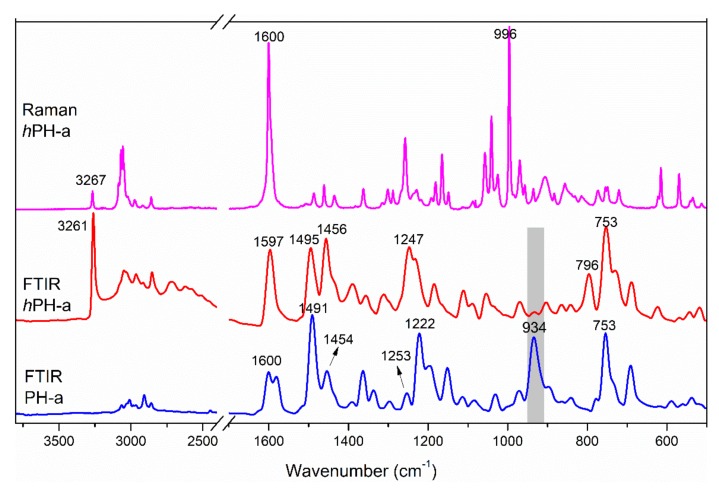

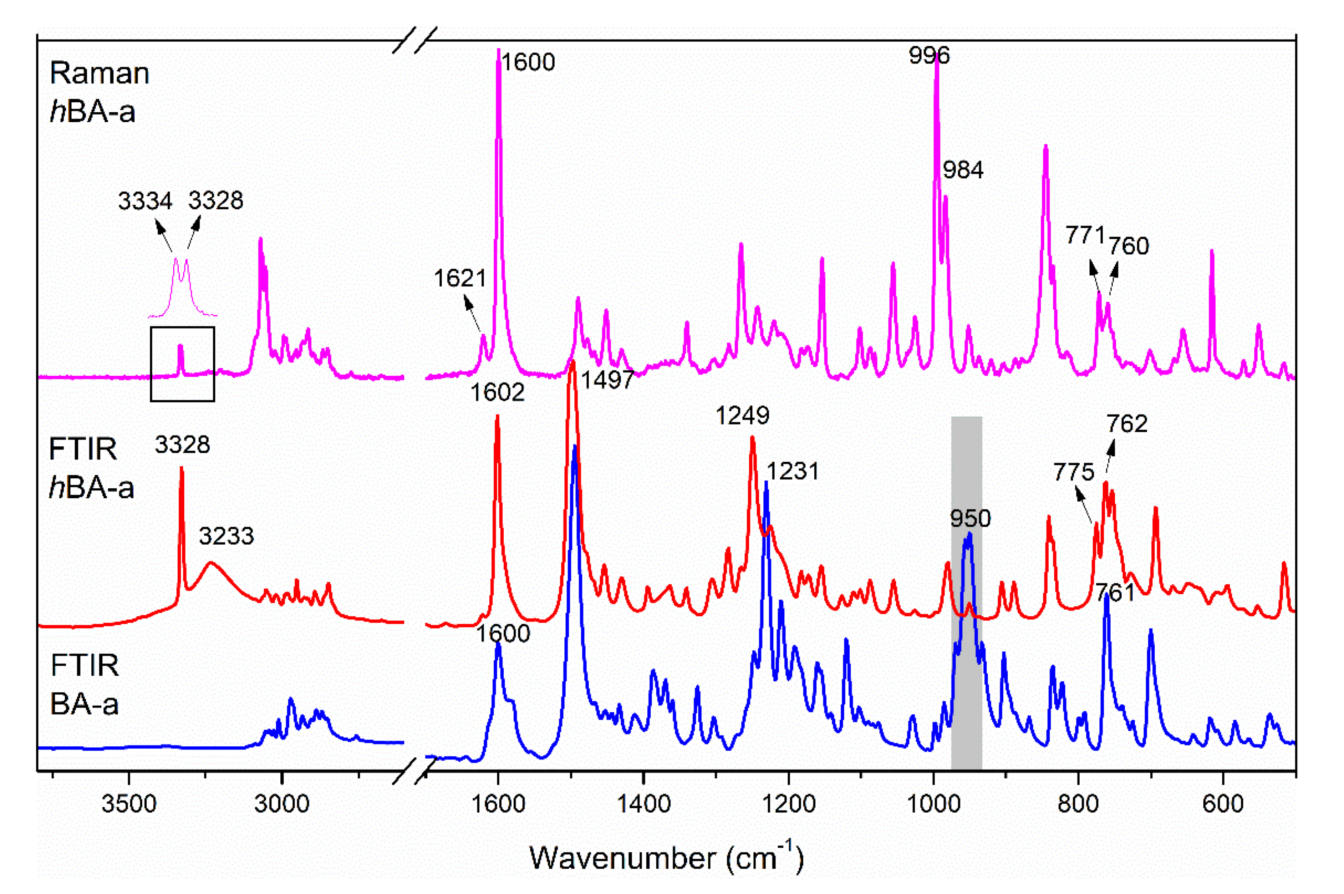
Raman and Fourier transform infrared (FTIR) spectra of *h*PH-a and PH-a, *h*BA-a and BA-a.

The molecular structures of *h*PH-a and *h*BA-a were also verified by FTIR and Raman spectroscopies, and the spectra of *h*PH-a, PH-a, *h*BA-a and BA-a are shown in Figure 2. The band assignments of the benzoxazine monomers and their opened structures are referred to in the reported results [43,44,45]. In the FTIR spectra, the 1600 cm^−1^ band of PH-a and 1597 cm^−1^ band of *h*PH-a are typical of aromatic skeletal vibrations, and the 1491 cm^−1^ band of PH-a and 1495 cm^−1^ band of *h*PH-a are attributed to the disubstituted benzene ring. The 753 cm^−1^ band is assigned to the Ar-H out-of-plane vibration. The bands at 1222 and 934cm^−1^ of PH-a correspond to the antisymmetric C-O-C stretching vibration and the O-C_2_ vibration in the oxazine ring with a minor contribution from the phenolic ring, respectively [45]. After hydrolysis, the 934 and 1222 cm^−1^ bands disappeared while a new band at 3261 cm^−1^ appeared in *h*PH-a, revealing that the oxazine ring was opened and the -OH and -NH groups were newly formed during the HCl treatment. The stretching vibration of -OH became broad and heavily overlapped with -CH. There are an additional very sharp bands at 3261 cm^−1^ for *h*PH-a and 3328 cm^−1^ for *h*BA-a that are suspected to be due to the NH stretching mode. The formation of the -NH group could be further confirmed by the new band at 796 cm^−1^, which is the characteristic peak of the N-H out-of-plane bending vibration. 

For *h*BA-a, its FTIR spectrum showed a similar phenomenon compared to BA-a. The 1231 and 950 cm^−1^ bands that are assigned for the antisymmetric C-O-C stretching vibration and the O-C_2_ vibration in the oxazine ring with a minor contribution from the phenolic ring, respectively, disappear, but three new bands at 3328, 3233 and 796 cm^−1^ appear in *h*BA-a, corresponding to the -NH stretching vibration, -OH stretching vibration and N-H out-of-plane bending vibration, respectively, after the HCl treatment.

The Raman spectra, as a complementary technique to FTIR spectroscopy, showed the typical peaks, as follows: the 1600 cm^−1^ line in both *h*PH-a and *h*BA-a corresponds to the monosubstituted benzene ring while 996 cm^−1^ is the vibration of the disubstituted and trisubstituted benzene rings in *h*PH-a and *h*BA-a, respectively. Corresponding to the very sharp band in the infrared spectra of *h*PH-a and *h*BA-a, the Raman spectra also show sharp lines near the same frequency. Generally, the oxazine ring breathing mode of benzoxazine shows a strong singlet peak at around 750 cm^−1^, but for both *h*PH-a and *h*BA-a, no strong peak is observed around 750 cm^−1^, indicating the opening of the oxazine ring, which is consistent with the results of the NMR and FTIR. 

An elementary analysis of *h*PH-a and *h*BA-a was performed; the results are shown in Table 1. The experimentally determined values are very similar to the theoretical values, indicating the high purity of both the intermediates obtained.

**Figure 3:**
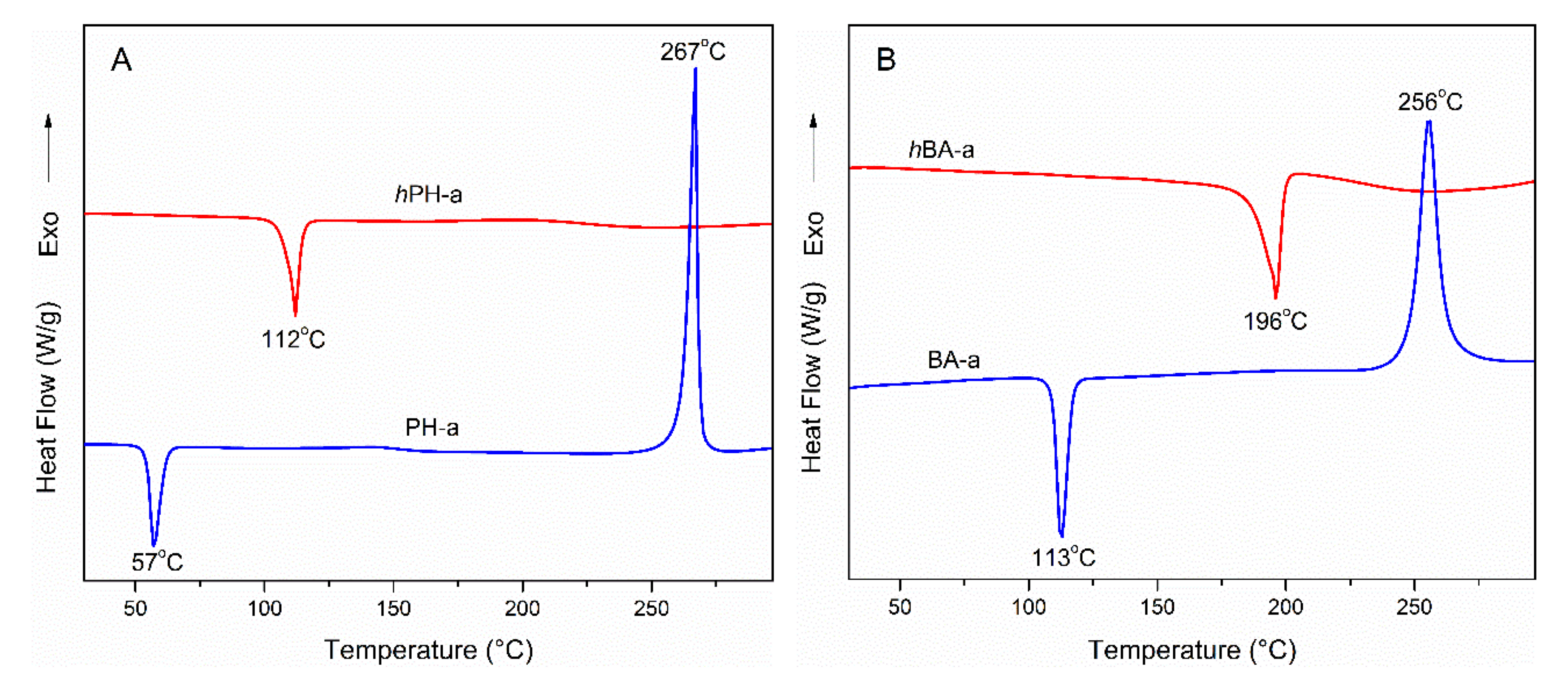
Differential scanning calorimetry (DSC) thermograms of *h*PH-a and PH-a (**A**), *h*BA-a and BA-a (**B**).

The melting and polymerization behaviors of the original benzoxazine and its intermediates were studied by a DSC study; the thermograms are shown in Figure 3. PH-a and BA-a show sharp melting endotherms at 57 and 113 °C and ring-opening polymerization exotherms at 267 and 256 °C, respectively. It is noteworthy that the melting endotherms at 112 °C of *h*PH-a and 196 °C of *h*BA-a are much higher than those of PH-a and BA-a, respectively, possibly due to the presence of intermolecular hydrogen bonds in the crystals. The high melting peaks also indicate that these intermediates could remain stable as independent compounds in a broad temperature range, and thus can be used as a raw material for further synthesis of various benzoxazine monomers, as shown in this paper. The polymerization exotherms of PH-a and BA-a disappear in *h*PH-a and *h*BA-a, indicating their inability to polymerize. There is an extremely weak and broad exotherm around 200 °C in the thermograms of both *h*PH-a and *h*BA-a. While the nature of this exotherm is not known at this time, it is possible that it could be due to the bond cleavage of the aniline unit. 

**Figure 4:**
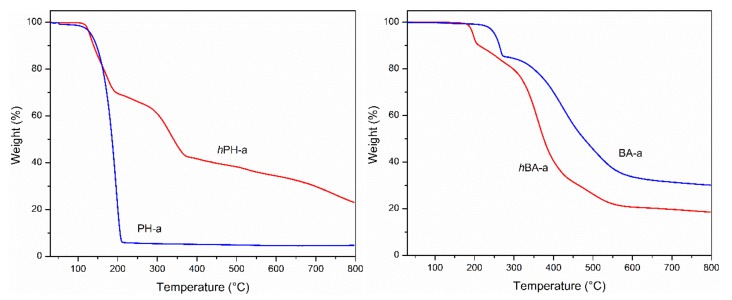
TGA curves of *h*PH-a and PH-a, *h*BA-a and BA-a.

The thermal decomposition behavior is characterized by the TGA in Figure 4. Both *h*PH-a and *h*BA-a show significant weight reductions at around 200 and 350 °C. Since the molecular weight of *h*PH-a and *h*BA-a are significantly different from each other, the weight reduction that coincides at around 200 °C is due not likely to the simple evaporation of the entire molecule but rather the fragmentation of the same type, again likely to be the aniline unit.

**Figure 5:**
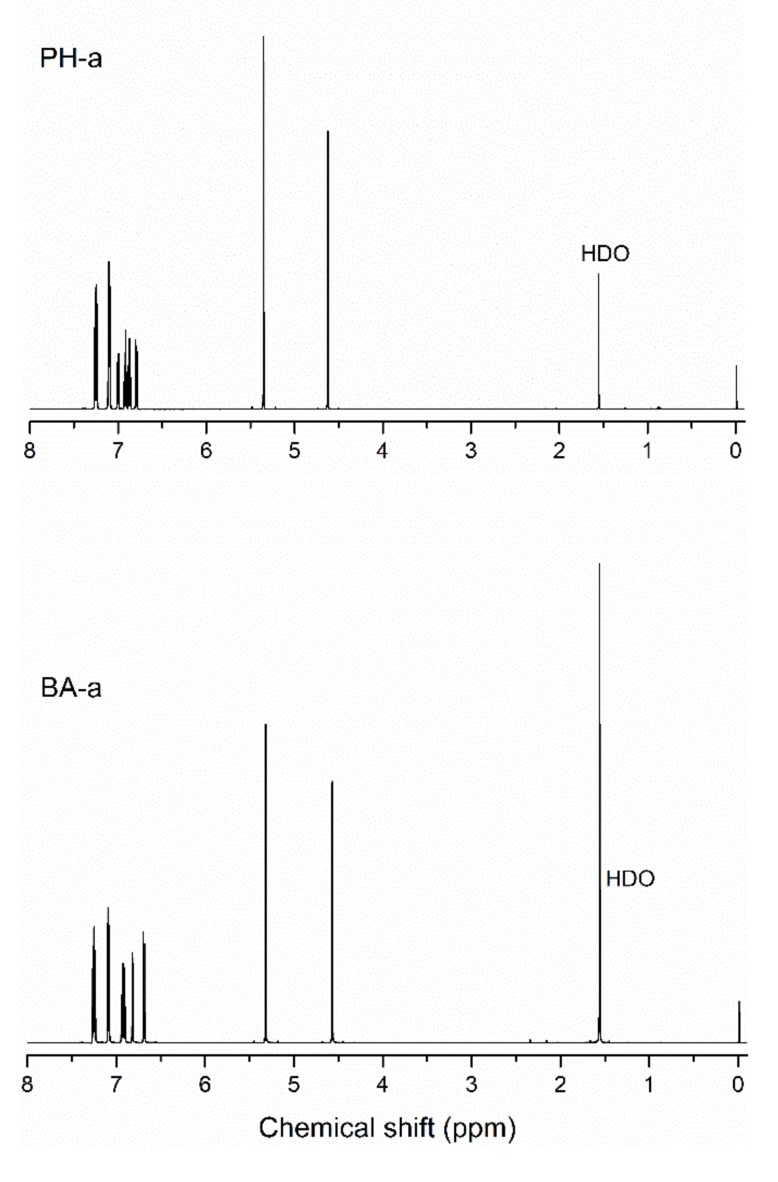
NMR spectra of PH-a and BA-a from 2-(aminomethyl)phenol.

From the above analyses, the pure and stable 2-(aminomethyl)phenols, *h*PH-a and *h*BA-a, were obtained. In order to demonstrate the feasibility of using these compounds as independent reactants, we used them to react with paraformaldehyde. From the ^1^H NMR in Figure 5, resonances at 4.62 (Ar-CH_2_-N) and 5.35 ppm (O-CH_2_-N) of PH-a, and resonances at 4.57 (Ar-CH_2_-N) and 5.32 ppm (O-CH_2_-N) of BA-a, show that PH-a and BA-a are clearly re-synthesized from these 2-(aminomethyl)phenols, demonstrating the reversible nature of these procedures. 

**Scheme 2:**
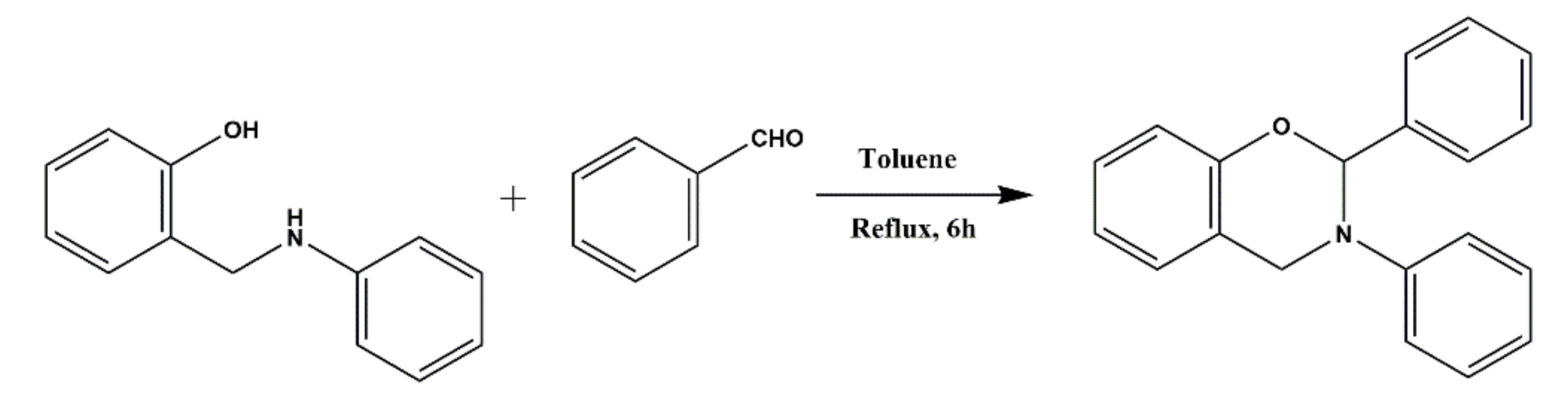
Preparation of PH-a-[*2*]*ba* from *h*PH-a and benzaldehyde.

**Figure 6:**
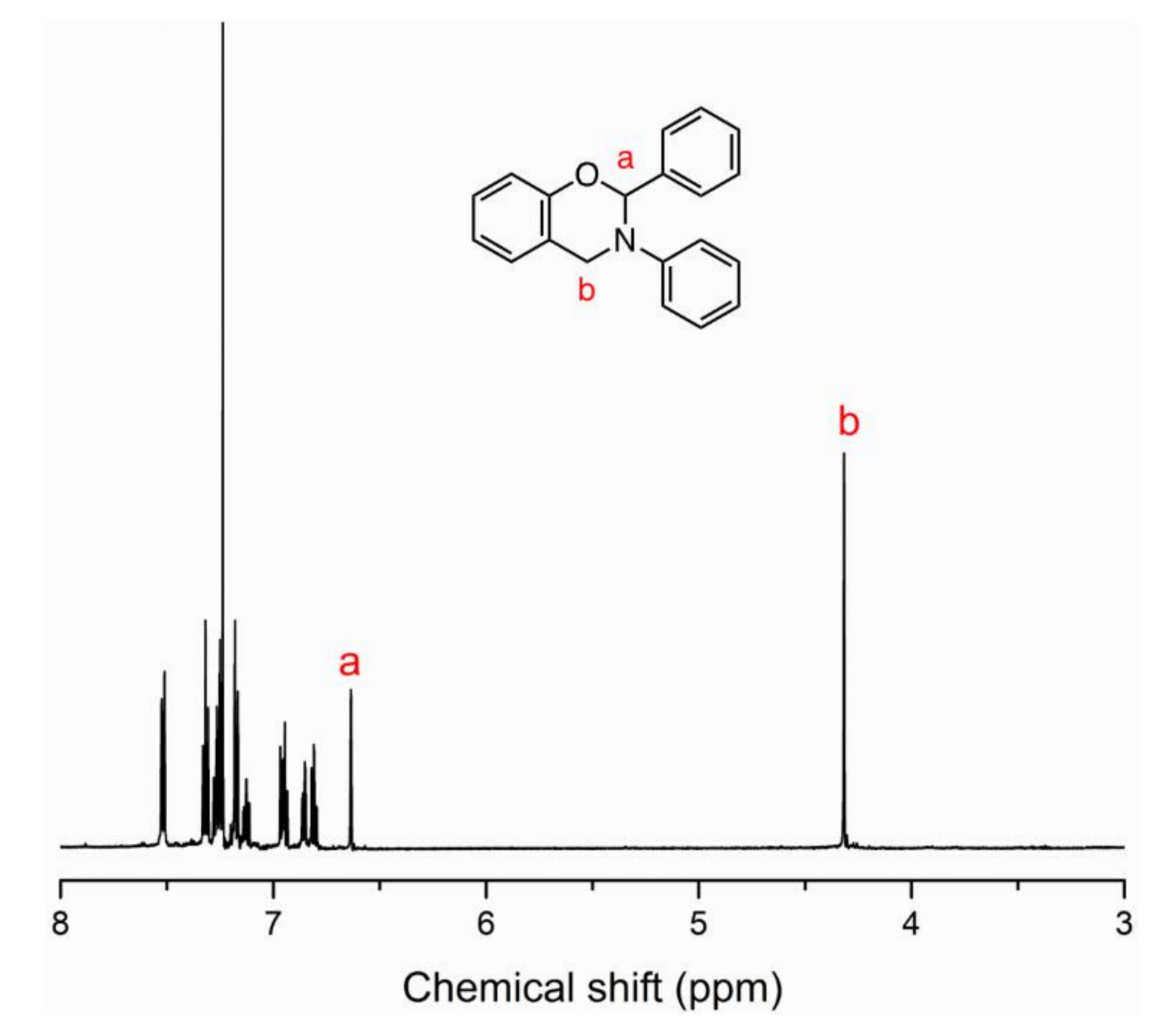
^1^H NMR spectrum of PH-a-[*2*]*ba.*

However, the hydrolyzed and purified benzoxazine are not only able to obtain the original benzoxazines. A more useful application might be to use other aldehydes rather than formaldehyde to obtain the 2-substituted benzoxazine monomer, as shown in Scheme 2 and Figure 6, where benzaldehyde is used to obtain a phenyl substituent at the 2-position of the oxazine ring. Since formaldehyde is carcinogenic, the use of other aldehydes that are less toxic is highly desirable. Furthermore, being able to use aldehydes other than formaldehyde will allow for the possibility of synthesizing many more benzoxazines, further expanding the horizon of the ability to tailor the desired properties. The ^1^H NMR spectrum clearly shows the successful synthesis of the target compound. The Ar-CH_2_-N and O-CHAr-N resonances are located at 4.31 and 6.63 ppm, respectively. These data are consistent with the previously reported PH-a-[*2*]*ba*, indicating the success of the HCl hydrolysis method for obtaining the same intermediates as traditional methods. 

Traditionally, this type of oxazine-substituted benzoxazine was synthesized by the three-step method, first starting from a phenolic compound with an aldehyde group at the ortho position. This was reacted with a primary amine to obtain a Shiff base, which was then reduced by NaBH_4_ to obtain the saturated 2-(aminomethyl)phenol, the compound of interest in this paper. This three-step process is unfavorable as the availability of the starting ortho-aldehyde-substituted phenol is highly limited, and the use of NaBH_4_ is expensive and needs care during synthesis. We have now demonstrated that exactly the same compound can be obtained in these more environmentally friendly conditions, which broadens the availability of the starting compounds to a vast number of compounds so long as the benzoxazine monomer can be obtained, and the process can proceed at a high yield. Such an oxazine-ring-substituted benzoxazine has been reported to undergo similar anionic ring-opening polymerization as the well-studied, non-oxazine-ring-substituted 1,3-benzoxazines [37]. Although only one example is shown here to demonstrate the feasibility, the same approach can be taken to synthesize the bis-functional monomers and multi-functional monomers, including main-chain, side-chain, telechelic, branched, hyperbranched, and dendrimer-type benzoxazines, into oxazine-ring-substituted benzoxazines. In other words, all benzoxazines synthesized using the standard Mannich condensation approach can also be made into the oxazine-ring-substituted compounds using the currently reported methodology.

## 4. Conclusions

The typical commercialized benzoxazines of PH-a and BA-a have been hydrolyzed by HCl to open their oxazine rings to obtain pure 2-(aminomethyl)phenol derivatives for the first time. The results obtained from the NMR, FTIR, and Raman spectroscopies, and elementary analysis strongly support the opening of the oxazine ring and successful isolation of the hydrolyzed intermediates that have stability in a broad temperature range. In addition, analyses from the DSC and TGA studies indicate their stability to store as independent compounds. The hydrogen bonding between the -OH and -NH endow a high melting temperature for them. Further, the acquisition of PH-a, BA-a and PH-a-[*2*]*ba* from the 2-(aminomethyl) phenolic derivatives reflects the feasibility of synthesizing the novel 1,3-benzoxazine with a functional group on the 2-position of the oxazine ring, which would enrich the polymerizable benzoxazines. 

## Figures and Tables

**Table 1:** Elementary analysis of intermediates.

	C		H		N	
	Theoretical	Experimental	Theoretical	Experimental	Theoretical	Experimental
*h*PH-a	78.39%	78.26%	6.53%	6.51%	7.04%	6.87%
*h*BA-a	79.45%	79.33%	6.85%	6.57%	6.39%	6.09%
